# Genome sequencing of 39 *Akkermansia muciniphila* isolates reveals its population structure, genomic and functional diverisity, and global distribution in mammalian gut microbiotas

**DOI:** 10.1186/s12864-017-4195-3

**Published:** 2017-10-18

**Authors:** Xianfeng Guo, Shenghui Li, Jiachun Zhang, Feifan Wu, Xiangchun Li, Dan Wu, Min Zhang, Zihao Ou, Zhuye Jie, Qiulong Yan, Peng Li, Jiangfeng Yi, Yongzheng Peng

**Affiliations:** 1 Department of Laboratory Medicine, Zhujiang Hospital, Southern Medical University, Guangzhou, 510282 China; 2 Shenzhen Puensum Genetech Institute, Shenzhen, 518052 China; 3 Department of Biochemistry and Molecular Biology, Dalian Medical University, Dalian, 116044 China; 4 Guangzhou Kangze Medical Science and Technology Co., Ltd, Guangzhou, 510630 China

**Keywords:** *Akkermansia muciniphila*, Genome diversity, Population structure, Mammalian gut microbiota, Antibiotic resistance

## Abstract

**Background:**

*Akkermansia muciniphila* is one of the most dominant bacteria that resides on the mucus layer of intestinal tract and plays key role in human health, however, little is known about its genomic content.

**Results:**

Herein, we for the first time characterized the genomic architecture of *A. muciniphila* based on whole-genome sequencing, assembling, and annotating of 39 isolates derived from human and mouse feces. We revealed a flexible open pangenome of *A. muciniphila* currently consisting of 5644 unique proteins. Phylogenetic analysis identified three species-level *A. muciniphila* phylogroups exhibiting distinct metabolic and functional features. Based on the comprehensive genome catalogue, we reconstructed 106 newly *A. muciniphila* metagenome assembled genomes (MAGs) from available metagenomic datasets of human, mouse and pig gut microbiomes, revealing a transcontinental distribution of *A. muciniphila* phylogroups across mammalian gut microbiotas. Accurate quantitative analysis of *A. muciniphila* phylogroups in human subjects further demonstrated its strong correlation with body mass index and anti-diabetic drug usage. Furthermore, we found that, during their mammalian gut evolution history, *A. muciniphila* acquired extra genes, especially antibiotic resistance genes, from symbiotic microbes via recent lateral gene transfer.

**Conclusions:**

The genome repertoire of *A. muciniphila* provided insights into population structure, evolutionary and functional specificity of this significant bacterium.

**Electronic supplementary material:**

The online version of this article (10.1186/s12864-017-4195-3) contains supplementary material, which is available to authorized users.

## Background

*Akkermansia muciniphila* is a commensal anaerobe that is found to reside in the intestinal tract of more than 80% of human population [1–3], constituting 1–4% of the total bacterial cells in the healthy adult feces. The presence of *A. muciniphila* is also detected in feces from newborns and the incidence increases rapidly during the first year of life [4], reaching 0.9% and 1.6% of relative abundance in 6- and 12-month-old infants, respectively [5]. Specifically, *Akkermansia*-like organisms are widely distributed in the intestines of non-human mammals such as lemur, gorilla [6] and mice [7], as well as other vertebrates such as chickens [8] and zebrafish [9].

When residing on the mucus layer of intestinal tract [10], *A. muciniphila* uses intestinal mucins, the highly glycosylated proteins of the epithelial mucus layer, as its major carbon and nitrogen sources that leads to the production of acetate and propionate, which are the important energy sources of human intestine epithelial cells [10]. This unique mucin-degrading feature makes *A. muciniphila* a modulator for gut homeostasis via strengthening the integrity of the epithelial cell layer [11] and regulating the gut barrier function [12, 13]. Recent studies demonstrated that *A. muciniphila* is beneficial to host health; e.g., *A. muciniphila* treatment reversed high-fat-diet-induced obesity and metabolic disease in mice [14, 15], and an increase in the *Akkermansia* spp. population induced by metformin treatment improved glucose homeostasis in mice [16]. Moreover, growing evidences showed that the abundance of *A. muciniphila* was inversely correlated with body weight [17, 18], type 1 diabetes [19], inflammatory bowel disease [20], and autism [21], in both mice and humans. Other studies found that *A. muciniphila* was enriched in type 2 diabetes [22] and colorectal cancer subjects [23].

The first and only available *A. muciniphila* genome, ATCC BAA-835, was sequenced in 2011 [24], comprising one circular chromosome of 2.66 Mbp. This genome showed distinct phylogenetic features in contrast with other genomes of the Verrucomicrobia phylum, as only 29% of genes were shared with its closest relative, varying largely in G + C content and genome size [24]. Specifically, when comparing all available bacterial or archaeal genomes in the national center for biotechnology information (NCBI) database, no non-*Akkermansia* sequences hit more than 90% of nucleotide identity with the whole *A. muciniphila* genome, indicating a unique and conservative evolutionary status of this bacterium [24] (we confirmed this result in July 2017 with 23,802 sequenced genomes). These evidences suggest that *A. muciniphila* has independently evolved for a long period, during which at least five divergent clades emerged, as revealed by phylogenetic analysis based on *Akkermansia* 16S rRNA gene sequences from mammalian-derived samples [8]. Furthermore, in our previous study [25], we isolated 22 *A. muciniphila* strains from human fecal samples and revealed 12 distinct subtypes via DNA fingerprint analysis. This motivated us to build the genome repertoire of *A. muciniphila* and investigate its population structure and characteristics on genomic level.

Here, we whole-genome shotgun sequenced and analyzed 39 new *A. muciniphila* strains isolated from human (*n* = 33) and mouse (*n* = 6) fecal samples. We also reconstructed 106 newly *A. muciniphila* draft genomes from extensive available metagenomic datasets of human (including Chinese, European and American), mouse and pig gut microbiomes. Our results showed that the genome contents of *A. muciniphila* are flexible with an open pangenome and frequently acquire genes from other bacteria via recent lateral gene transfer (LGT). We revealed a remarkably high genetic diversity within *A. muciniphila* and accordingly classified this species into three species-level phylogroups. Based on this enormous amount of genomic data, we accurately quantified the occurrence rate and abundance of *A. muciniphila* phylogroups in mammalian gut microbiomes, and investigated its association with body mass index and anti-diabetic drug usage in human subjects. Our results thus provided the genomic and evolutionary landscape of *A. muciniphila*.

## Results

### Overview of the *A. muciniphila* genomes

A total of 39 *A. muciniphila* isolates were collected and whole-genome shotgun sequenced by Illumina approach. De novo assembly of their genomic data revealed varying genome size ranging from 2.65 to 3.20 Mbp (averaging 2.86 Mbp, Additional file 1: Table S1). The genome sizes of almost all (38/39) isolates were larger than that of ATCC BAA-835, indicating that extensive genomic contents of *A. muciniphila* were unexplored previously.

**Fig. 1:**
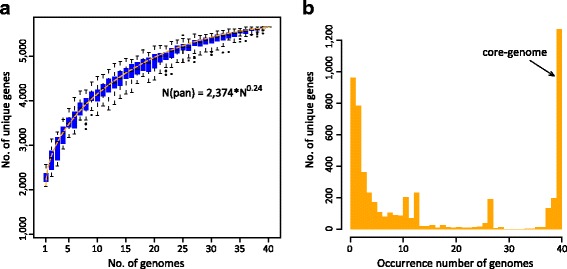
Pangenome, core-genome and gene occurrence of the 40 analyzed *A. muciniphila* isolates. (**a**) Pangenome accumulation curves. The blue boxes denote the number of unique genes discovered with the sequential addition of new genomes. The equations below the graph show parameters for fits to Heap’s law, and the positive exponent indicates an open state of the pangenome. (**b**) Gene occurrence plot shows the core-genome and additional accessory genes of *A. muciniphila*

The number of protein-coding genes of the 40 available *A. muciniphila* genomes (39 newly isolates and strain ATCC BAA-835) varied from 2138 to 2664 (averaging 2370, Additional file 1: Table S1). To assess the gene contents of *A. muciniphila*, we identified a pangenome containing 5644 unique protein-coding genes among these genomes. The gene accumulation curve fit the Heap’s low $\left(n=\kappa N\gamma \right)$ with parameters γ = 0.25 ± 0.02 (95% confidence interval) (Fig. 1a), and the occurrence of new genes fit the power low $\left(n\sim N\hbox{--} \alpha \right)$ with exponent α = 0.70 ± 0.04 (Additional file 2: Figure S1). Both findings indicate an open pangenome of *A. muciniphila*. 34 unique genes will potentially be added along with the availability of additional genomes. The gene occurrence plot (Fig. 1b) showed a core-genome containing 1275 genes that were present in all sequenced strains, whereas the other genes were additional accessory genes that were mostly present in a few of genomes (60% of genes found in ≤5 genomes and one fourth found in only one genome).

### Population structure within *A. muciniphila* species

**Fig. 2:**
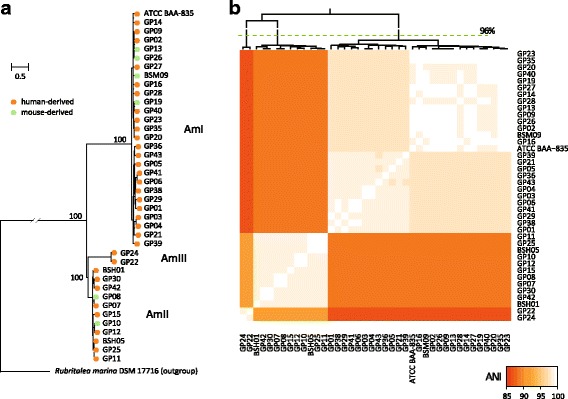
Phylogroups of *A. muciniphila*. (**a**) Maximum likelihood phylogenetic tree based on core genome SNPs of 40 *A. muciniphila* isolates. The tree is inferred using RAxML with 1000 replications, with *Rubritalea marina* DSM 17716 as outgroup species. Bootstrap support values are shown for the separation nodes of the main phylogroups. (**b**) Hierarchical clustering of isolates based on whole-genome level average nucleotide identity (ANI). Pairwise ANI between isolates is shown as a heat map

To investigate the population structure of *A. muciniphila*, we identified 270,928 high-quality single nucleotide polymorphisms (SNPs) within the core-genome (1.47 Mb of sequences in total). Such a huge number of SNPs indicated the highest level of nucleotide diversity (184 SNPs kb^−1^) in *A. muciniphila* compared with other prevalent human gut microbial species [26], which may be facilitated by ancient evolutionary history and large population symbiotic in human and other mammals (see Discussion). Maximum likelihood (ML) phylogenetic analysis of these SNPs (Fig. 2a) identified three major phylogroups (defined as AmI, AmII and AmIII), with 100% bootstrap support. This separation was also consistent with average nucleotide identity (ANI) clustering and principal components analysis (PCA) based on the whole genomic data (Fig. 2b and Additional file 2: Figure S2). Within each phylogroup, the ANI between genomes was 97.2–100%, whereas ANI between phylogroups was 86.8–91.5%. Based on the between-phylogroup nucleotide conservation of 96%, which is normally used as a threshold for prokaryotic species definition [27], we noted that AmI, AmII and AmIII are distinct species and these phylogroups constitute discrete bacterial populations that are evolving independently. Conversely, these phylogroups also shared highly consistent phenotypic characteristics [25], habitat, and conservative 16S rRNA genes (nucleotide similarity >99% between any two genomes).

**Fig. 3:**
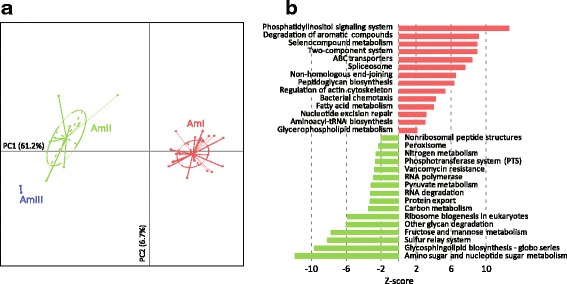
Functional difference between *A. muciniphila* phylogroups. (**a**) Principal components analysis based on the KEGG profile. Isolates on the first and second principal components (PC1 and PC2) are plotted by nodes. Lines connect isolates in the same groups, and colored circles cover the isolates near the center of gravity for each group. (**b**) The significantly different pathways between AmI and AmII/III. Pathways with false discovery rate (FDR) corrected *P* value (*q*) of <0.05 (converted to Z-score; two-tailed Fisher’s exact test) are shown

To examine the functional significance, we annotated the *A. muciniphila* pangenome using the eggNOG (evolutionary genealogy of genes: Non-supervised Orthologous Groups) [28] and KEGG (Kyoto Encyclopedia of Genes and Genomes) [29] databases. Due to the lack of extensive functional research on *Akkermansia* species or its evolutional related taxa, only 29.6% (1668/5644) genes could be assigned into provisional function, and the others were mostly “hypothetical proteins”. All *A. muciniphila* strains carried a large proportion of proteins that are involved in genetic information processing (translation, DNA replication and repair), membrane transport, signal transduction and metabolism of a variety of compounds (Additional file 2: Figure S3). However, each phylogroup carried a unique subset of proteins when comparing their functional profiles. Based on the PCA analysis of KEGG pathways (Fig. 3a), a dramatic divergence was found between the genomes of phylogroup AmI and AmII/AmIII, whereas AmII and AmIII were relatively closer, possibly due to their evolutionary relationship. The AmI genomes were enriched in “phosphatidylinositol signaling system”, “degradation of aromatic compounds”, “two-component system”, “ABC transporters”, etc.; while the AmII/AmIII genomes were enriched in “amino sugar and nucleotide sugar metabolism”, “glycosphingolipid biosynthesis - globo series”, “sulfur relay system”, “fructose and mannose metabolism”, etc. (Fig. 3b). *A. muciniphila* is commonly known as a mucin-degrading bacteria in the human fecal microbiota, likely depending on its possession of abundant extracellular glycosidases [24]. When we annotated the carbohydrate-active enzymes (CAZymes) on the *A. muciniphila* genomes, AmI also showed substantial difference from that of AmII and AmIII (Additional file 2: Figure S4). Generally, the AmII/AmIII genomes carried larger number of CAZymes than AmI did (average number of CAZymes, 210 vs. 191; *P* < 0.001), especially the glycosyl transferase family 4 (GT4) (average number of genes, 35 vs. 22; *P* < 0.001) which is involved in the biosynthesis of several oligosaccharides. This result indicated that AmII and AmIII are more versatile in metabolizing carbohydrates and substrates, which could also be reflected by the enrichment of polysaccharide (e.g. fructose and mannose) metabolism in KEGG pathways.

### Global distribution of *A. muciniphila* phylogroups in mammalian hosts

**Fig. 4:**
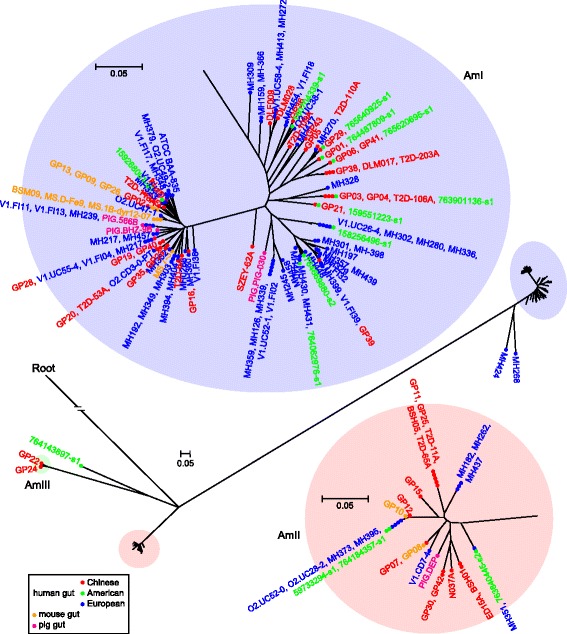
Phylogroups of *A. muciniphila* strains derived from human and other mammalian gut microbiotas. Maximum likelihood phylogenetic tree based on core-genome SNPs of 40 *A. muciniphila* isolates and 106 new constructed genomes (MAGs). The tree is inferred using RAxML with 1000 replications

To explore the genomic content of *A. muciniphila* in human and other mammalian gut microbiotas, firstly, public metagenomic datasets used in the cataloguing of gut microbiomes in human (1267 fecal samples, from Chinese, *n* = 368 [22]; European, *n* = 760 [30]; and American, *n* = 139 [31]), mouse (184 samples [32]) and pig (290 samples [33]) were queried. Using a simplified approach based on the genomic unique of *A. muciniphila* (see Methods), we reconstructed 106 metagenome assembled genomes (MAGs) from these microbiomes (99 in human, 3 in mouse, and 4 in pig), and obtained an average of 2.82 Mbp (average contigs number of 235, average N50 length of 42.9 kbp) genome size for each MAG (Additional file 3: Table S2). Phylogenetic analysis of the MAGs was performed combined with 40 known *A. muciniphila* isolates. Based on the ML-tree (Fig. 4) and a nucleotide conservation threshold of 96%, the majority (103/106) of newly constructed genomes were assigned into the major *A. muciniphila* phylogroups, AmI and AmII. Of the other 3 unplaced MAGs, two (from European) were highly homologous to AmI and one (from American) was highly homologous to AmIII. Both AmI and AmII contained MAGs from three human populations, mouse and pig (Fig. 4), demonstrating that the distribution of *A. muciniphila* phylogroups were transcontinental and across mammalian hosts.

**Fig. 5:**
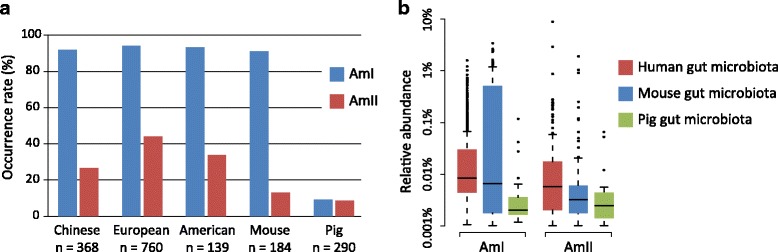
Distribution of *A. muciniphila* phylogroups in mammalian gut microbiomes. (**a**) Occurrence rate in the Chinese, European and American population and other mammalian gut microbiotas. (**b**) Relative abundance of *A. muciniphila* phylogroups in selected samples

Such enormous genomic data enabled us to accurately quantify the occurrence rate and abundance of *A. muciniphila* phylogroups in human and other mammalian gut microbiotas. Of the three phylogroups, we revealed that AmI was the most frequently occurring phylogroup which was found in 93% of all tested human population (minimum relative abundance >0.01% in metagenomic sample, and covering >10% of the *A. muciniphila* core-genome), 91% of mice and 9% of pigs (Fig. 5a). AmII was also frequently found in human gut, with higher occurrence rate in European (44%) than in Chinese (27%; *P* < 0.001, Fisher’s exact test) and American (33%; *P* = 0.02). However, AmII was less frequently found in mouse (12%; *P* < 0.001 compared to human microbiota) and pig (9%; *P* < 0.001) gut microbiotas. Comparing of relative abundance showed AmI had higher abundance in mouse gut than in human and pig, whereas AmII majorly resided in human gut (Fig. 5b).

### Abundance of *A. muciniphila* associated with obesity and anti-diabetic drug

Recent studies explored the relationship between *A. muciniphila* and obesity and other diseases [14, 18, 34]. In this study, we expanded the analysis of the correlation between relative abundances of *A. muciniphila* phylogroups (AmIII was not analyzed for its rare occurrence rate) and host phenotypes, including gender, age and body mass index (BMI), in the phenotype-available Chinese individuals. No significant correlation was found between gender, age, BMI and the relative abundance of *A. muciniphila* phylogroups (Additional file 2: Figure S5a-b). However, a significant decrease of *A. muciniphila* was found in individuals with BMI of over 30 (*P* < 0.01 for both AmI and AmII, Mann-Whitney U test), consistent with previous study that showed decrease of *A. muciniphila* in severely obese individuals [18]. We also validated the recent study [35] by revealing a significant enrichment of *A. muciniphila* in gut microbiota of the anti-diabetic metformin treated individuals compared with the untreated diabetic or healthy individuals (*P* = 0.007, Mann-Whitney U test after adjusted gender, age and BMI; Additional file 2: Figure S5c). Specially, this phenomenon was only found in AmI.

### *A. muciniphila*’s acquisition of antibiotic resistance genes from co-ecological species

**Fig. 6:**
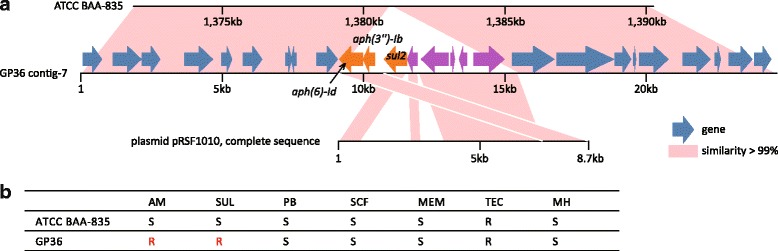
Acquisition of antibiotic resistance genes during *A. muciniphila* evolution. (**a**) *A. muciniphila* GP36 gained 8 genes from the plasmid pRSF1010. Genomic fragments painted with red denote their highly homologous regions (nucleotide similarity >99%). Genes are denoted by arrows, and the three antibiotic resistance genes are labeled. (**b**) Drug sensitive results of *A. muciniphila* GP36 and ATCC BAA-835. Definition of abbreviations: AM, amikacin; SUL, sulfonamides; TEC, teicoplanin; PB, polymyxin; SCF, cefoperazone-sulbactam; MEM, meropenem; MH, minocycline

As the only Verrucomicrobia bacteria resident in human and other mammalian guts, *A. muciniphila* showed drastic differences in morphology and life cycle compared with other Verrucomicrobia species [36, 37], which was assumed to be related with its distinct adaption during mammal-associated evolution. We assumed that the *A. muciniphila* strains gained genes from other species via lateral gene transfer during the evolutionary history mammalian gut. To validate this, we searched for the LGT events by comparing the full gene sets of 40 *A. muciniphila* strains (39 newly isolates and ATCC BAA-835) with all non-*Akkermansia* genomes from the NCBI database using BLAST (see Methods). Ten candidate transfer events involving 83 genes (Additional file 4: Table S3) were identified with threshold of more than 85% similarly with the nearest genomes. All of these transfer events were of low frequency (occurring in less than four strains) and located in the terminal of phylogenetic tree, indicating that LGT occurred recently. *A. muciniphila* acquired extra genes from a wide range of distinct taxa, including *Bacteroides* spp., *Bifidobacterium longum*, and several Firmicutes. Meaningfully, most of these taxa were associated with human gut habitats, suggesting that these genes were gained from the co-ecological species. As a striking example, an LGT event was observed in *A. muciniphila* GP36 which gained 8 genes from the plasmid pRSF1010 originally found in *Salmonella enterica* (Fig. 6a). 3 out of 8 genes were antibiotic resistance genes, namely *sul2* (sulfonamide-resistant dihydropteroate synthase), *aph(6)-Id* and *aph(3″)-Ib* (aminoglycoside phosphotransferase). Drug sensitive test validated the emergence of resistance against corresponding antibiotics (Fig. 6b). This event indicated that the *A. muciniphila* species might acquire antibiotic resistance genes via recent LGT to adapt to the high level of antibacterial gastrointestinal environment in modern lifestyle.

## Discussion

In vivo, *A. muciniphila* plays a crucial role in maintaining the integrity of the mucus layer, thereby reducing translocation of proinflammatory lipopolysaccharides and controlling adipose tissue metabolism, decreasing insulin resistance and keeping glucose homeostasis [14, 16]. Accumulating research evidences uncovered the beneficial effects of *A. muciniphila* in host [13, 38]. For example, administration treatment of *A. muciniphila* reversed diet-induced metabolic disorders in animal models [14, 15], suggesting its potential function in prevention or treatment of human obesity and other metabolic diseases. Though an increasing number of research reports focus on *A. muciniphila* (>160 papers by a PubMed research for “*Akkermansia muciniphila* [title/abstract]” in July 2017), however, only one *A. muciniphila* strain (ATCC BAA-835) is isolated and publicly available, due to its arduous colonization conditions in vitro [39]. Here, we analyzed 39 *A. muciniphila* strains isolated from human and mouse feces via our recently developed methodology [25], as well as 106 new MAGs assembled from metagenomic datasets of human, mouse and pig feces samples.

For the first time, we revealed the genomic diversity and population structure of *A. muciniphila*, and identified three phylogroups, AmI, AmII and AmIII, within *A. muciniphila*. 113 of 146 (77%) known genomes were assigned into AmI, while 28 (19%) were assigned into AmII, with two AmIII genomes and three unplaced genomes. Phylogroups show high genomewide nucleotide diversity and distinct metabolism and function profiles (Fig. 2 and Fig. 3). Strains within each phylogroup are highly similar in nucleotide sequences (ANI > 97.2% between any two genomes), whereas strains between phylogroups are divergent (ANI < 91.5%). When using the nucleotide conservation of 96% as a threshold for prokaryotic species definition [27], these phylogroups are likely to be defined as different species. However, there are no obvious mechanistic barriers to gene flow between AmI, AmII, and AmIII. Indeed, we observed a number of gene flow events and several recombination events between three phylogroups (Additional file 2: Figure S6), suggesting a possible gene exchange and homologous recombination. Thus, we leave the species definition of *A. muciniphila* for future experimental and/or phenotypic investigation. Moreover, previous study [8] had divided the *Akkermansia* phylogenetic tree (based on >400 full-length 16S rRNA gene sequences) into five clades, while the conceivable *A. muciniphila* lineages were assigned into two distinct clades, clade 1 (16S rRNA gene similarity with strain BAA-835, 97–100%) and clade 4 (95–99%). Although *A. muciniphila* strains in our study was widely distributed in global human, mouse and pig, the 16S rRNA gene sequences of all strains were quite conserved, suggesting that more *A. muciniphila* phylogroups are still undiscovered, especially in non-human animals.

In functional analysis, genes that encode CAZymes are of particular interested, as them are required to metabolize most of dietary polysaccharides [40]. *A. muciniphila* genomes carried average 198 CAZymes, exceeding the number of CAZymes per genome for most microbes in the human gut microbiota (e.g. members of Firmicutes, but less than Bacteroidetes) [40], highlighting its predominant role in glycan metabolism for the mammalian hosts. In comparison, the AmII/AmIII genomes carried larger number of CAZymes than AmI did, especially the GT4, which is involved in the biosynthesis of oligosaccharides such as sucrose and mannose. Parallelly, the AmII/AmIII genomes harbored more proteins which belong to the KEGG pathways “amino sugar and nucleotide sugar metabolism”, “fructose and mannose metabolism” and “carbon metabolism” (Fig. 3b). Combining of these observations suggested more versatile in metabolizing carbohydrates and substrates for AmII and AmIII. Thus, the functional specificity of *A. muciniphila* phylogroups would further correlate to their phenotypes or lifestyle, however, systematic investigations of key enzymes and functions might be helpful in the future.

*A. muciniphila* is widely distributed in intestinal tracts of the animal kingdom, and the *Akkermansia* genus was considered as an indigenous member of the microbiota resident in various animals [8]. Recent studies revealed that a remarkably low number of microbial genes (~15,000) was shared in the gut microbiomes of human (representing 9.9 million non-redundant genes) [30], mouse (2.6 million) [32] and pig (7.7 million) [33]. Strikingly, one third (~5000) of these genes belonged to *A. muciniphila*, indicating that *A. muciniphila* is a major species that exists in multiple mammalian hosts. In our datasets, though the *A. muciniphila* was detected in 91% of mouse fecal samples (including all the samples collected from different providers from Europe, China and America [32]), only three MAGs all belonging to the major phylogroup AmI, were identified (Fig. 4), and the others were almost identical to one of them. This demonstrates that the genomic diversity of *A. muciniphila* in mouse gut microbiota is significantly lower than that in human, probably due to the similar genetic background and laboratorial environment of the mice [32].

During the evolution history, *A. muciniphila* presents a large proportion of losses of orthologous groups compared with its ancestral genome of the Verrucomicrobia phylum [24] (see Additional file 2: Figure S7 for the phylogenetic tree based on the available species of the Verrucomicrobia phylum and the type strains of *A. muciniphila*), which was speculatively due to its distinct adaption during host-associated evolution. In this study, we found yet another mechanism of *A. muciniphila* to adapt the mammalian gut habitat. Several *A. muciniphila* strains acquired extra genes from a wide range of microbial taxa, such as *Bacteroides* spp., *Lachnospiraceae* spp., and *Bifidobacterium longum*. Most of the genes are inside a transposable element that can be transferred between multiple species. A striking example is the acquisition of three antibiotic resistance genes (*sul2*, *aph(6)-Id* and *aph(3″)-Ib*) in strain GP36 (Fig. 6). These genes are part of the plasmid RSF1010, an 8684 bp broad host range plasmid that can replicate in most Gram-negative bacteria and Gram-positive Actinomyces [41, 42], and can simultaneously be found within integrative conjugative elements or chromosomal genomic islands in both Gram-positives and Gram-negatives [43, 44]. We assume that the acquiring of these genes is required to adapt to the high level antibiotic environments in current human gut.

Based on a recent research, *A. muciniphila* showed an inverse correlation with body weight in both mouse and human models [15, 18]. We expanded these observations in the Chinese cohorts and further found that both of the two major phylogroups of *A. muciniphila*, AmI and AmII, were associated with obesity (Additional file 2: Figure S5b). Previous studies found that anti-diabetic metformin treatment improved glucose homeostasis in association with increased *Akkermansia* spp. population in the gut microbiota of mice [16]. Likewise, metformin is also associated with higher relative abundance of *A. muciniphila* in the human gut [35]. In this study, we found only AmI was associated with metformin treatment in the Chinese diabetic individuals (Additional file 2: Figure S5c), suggesting a different anti-diabetic effect of AmI and AmII. In spite of these findings, more experimental evidences are required.

## Conclusions

This study characterized the genomic architecture of *A. muciniphila* based on whole-genome sequencing of 39 isolates and metagenomic-reconstructing of 106 draft genomes from mammalian gut feces. The genome contents of *A. muciniphila* are flexible with an open pangenome and frequently acquire genes from co-ecological bacteria via lateral gene transfer. We revealed high genetic diversity of *A. muciniphila* and classified them into three species-level phylogroups. We also quantified the occurrence rate and abundance of *A. muciniphila* phylogroups in mammalian gut microbiomes, and investigated its association with host phenotypes. In summary, our results demonstrated the notable population genomic diversity, functional specificity, geographically distribution and ecological adaptation of *A. muciniphila*. The comprehensive genomic framework of *A. muciniphila* provide solid foundation and practical support for future studies.

## Methods

### Bacterial isolates

Six strains were isolated from mouse fecal samples while other 34 strains (including A. muciniphila sp. GP37) were isolated from human feces. All 40 strains were isolated as previous described with some modifications [10]. Briefly, about 1.0 g of fresh feces were collected from mouse (C57BL/6) or outpatients in Zhujiang Hospital and mixed in 5.0 ml 0.9% salt solution thoroughly. The suspension was then collected for serial dilutions (10-fold) in 0.9% salt solution. 1.0 ml of the highest diluted suspension (10^−6^ dilution) was inoculated in 9.0 ml mucin medium and then the mixture was kept in anaerobic condition at 37 °C for about one week until obvious turbidity was observed. The mucin medium was made according to the method previously described [10]. The positive tubes after enrichment were further purified by repeated inoculation on the same medium containing 0.75% agar (OXOID, England). Single colonies were collected and identified by 16S rRNA gene sequencing.

### DNA preparation and sequencing

Bacteria were grown in mucin medium for 48 h and harvested by centrifuge at 400 g for 10 min. Bacterial DNA was extracted by Ampure Microbial DNA Kit (Magen, Guangzhou, China) according to maufacturer’s instruction. DNA library was constructed using the TruSeq DNA protocol LT Sample Perp Kit following the manufacturer’s instruction, and whole-genome shotgun sequenced using the Illumina HiSeq 2500 instrument, which generated a series of 150 bp paired-end reads with expected 250-300 bp insert size for every strain. High quality reads were extracted from the raw Illumina data by trimming the low quality (Q < 30) bases on the end of reads and filtering ‘N’-containing, adapter contamination or short length (< 100 bp) reads, using the FASTX-Toolkit [45].

**Genome assembly, annotation and pangenome analysis.** Short reads for each *A. muciniphila* isolate were assembled using Velvet [46], an algorithm for de novo short read assembly using *de Bruijn* graphs. For each isolate, the procedure was run multiple times using different *kmer* parameter ranging from 35 to 145 to generate the best assembly result. Then, the raw assembled genome was performed by contig extension and scaffolding by SSPACE [47], and the results were performed by gap closing procedure using GapFiller [48]. The shortest scaffolds were filtered with minimum length threshold of 200 bp. The previous finished genome of strain ATCC BAA-835 was downloaded from NCBI bacterial genome database (accession no. NC_010655).

Genomic annotation was implemented using the Prokka [49] pipeline. Prokka used a suite of prediction tools to identify the coordinates of genomic features within contigs, including small rRNA (5S, 16S and 23S rRNA) using RNAmmer [50], tRNA using Aragorn [51], and Clustered Regularly Interspaced Short Palindromic Repeats (CRISPR) using MINCED [52]. Prokka annotated the protein coding genes in a hierarchical manner: 1), Prodigal [53] identifies the coordinates of candidate genes; 2), the homologous proteins of *A. muciniphila* ATGG BAA-835 was used as trusted annotate from; and 3), the other protein families were searched from bacterial proteins in UniProt and RefSeq databases. After running Prokka, genes were further annotated to eggNOG (evolutionary genealogy of genes: Non-supervised Orthologous Groups, v4.5) [28] and KEGG (Kyoto Encyclopedia of Genes and Genomes, downloaded Feb. 2016) [29] databases using BLASTP (identity threshold of 35%, covering >70% of the gene length; −qcov_hsp_perc parameter, 0.7). Remaining genes with no matches in Prokka, eggNOG or KEGG were labeled as “hypothetical protein”. Glycoside hydrolase genes were annotated using the Carbohydrate-Active enZYmes (CAZy) [54] database.

Pangenome analysis was performed using Roary [55] with default parameters.

### Average nucleotide identity

Average nucleotide identity (ANI) between two genomes was calculated using the “ANIb” algorithm which uses BLAST as the underlying alignment method [27, 56].

### SNP calling and phylogenetic analysis

To detect SNPs in the core genome, paired-end reads of each isolate were mapped using BWA [57] (−n parameter, 22; minimal identity of 85%) against the reference genome of strain ATCC BAA-835. Mapped were considered only if they matched the core gene regions (1298 genes in 1.95 Mb of sequences) of the reference. Candidate variants were extracted using SAMtools mpileup [58], followed by BCFtools from the SAMtools package with filtering parameters of variant quality score greater than 50 and mapping quality greater than 30. Uncertain variant was further filtered if its major allele frequency (as calculated by BCFtools) is less than 80% or reads supporting number less than 5. Phylogenetic reconstruction was carried out using the maximum-likelihood program RAxML v8.2.4 [59] with a GTR model of evolution, and visualized using MEGA [60]. Robustness of the phylogenetic tree was estimated by bootstrap analysis in 1000 replicates.

### Metagenomic data source, draft genome reconstruction and further analysis

To analyze the distribution of *A. muciniphila* in human and other mammalian gut microbiomes three public metagenomic datasets were included: (i) 1267 human fecal samples provided by Li, et al. [30], which included 368 Chinese subjects (first published in [22], NCBI accession no. SRA045646 and SRA050230), 760 European subjects, and 139 American subjects (first published as part of the Human Microbiome Project [31], data available at http://www.hmpdacc.org/HMASM/); (ii), 184 mouse fecal samples [32]; and (iii), 290 pig fecal samples (287 in ref. [33], and 3 samples collected from a swine farm in China). All of these samples were performed DNA preparation, library preparation and whole-metagenome shotgun sequencing using the metagenomic-specific protocols, as detailedly described at [30–33], and all of the raw sequencing data were available in the GigaScience Database (http://www.gigadb.org/). Metagenomic samples were basically processed (e.g. high-quality reads extraction and host DNA contamination removing) using the MOCAT pipeline [61] under the bioinformatic platforms at Beijing Genomics Institute (BGI)-Shenzhen.

Metagenomic reads were assembled using MEGAHIT (a de novo assembler for large and complex metagenomic sequences) [62]. Based on highly conservative of *A. muciniphila* genomes, we therefore extracted putative *A. muciniphila* fragments from the assembled contigs of each metagenomic sample via aligned to the 40 known *A. muciniphila* genomes, using BLASTN search (identity >85%, align length ≥ 300 bp, and covering >70% of the contig length; −qcov_hsp_perc parameter, 0.7). Notably, the putative *A. muciniphila* sequences from one sample might be derived from two or more *A. muciniphila* strains. To avoid this, for each sample, the read depths for all putative *A. muciniphila* fragments were calculated, and only samples with unimodal read depth distribution of these fragments (indicating that only one major *A. muciniphila* strain exists in that sample) were retained. Low depth (<15X) and inconsistent (>3σ) fragments on each remaining sample were further removed. Lastly, we obtained 106 unimodal putative *A. muciniphila* genomes with minimum read depth of 15X from 106 metagenomic samples. The completeness of the draft *A. muciniphila* genomes was estimated by comparing to the known *A. muciniphila* core-genome (see Results), and all of these genomes obtained satisfactory assembling completeness (>95%).

The *A. muciniphila* reads were extracted from 106 metagenomic samples via reads mapping to the corresponding *A. muciniphila* MAG (using BWA, identity >95%), and were used for SNP calling based on the before-mentioned method. The accurate quantify of *A. muciniphila* in human and other mammalian gut microbiomes was preformed via reads mapping to the available genomes (all 146 strains) using BWA (identity >95%).

### Identification of lateral gene transfer

For each isolates, the LGT gene was identified via comparing its genes to all external *Akkermansia* genomes from the NCBI database using BLASTN (identity threshold of 85%, covering >90% of the gene length; −qcov_hsp_perc parameter, 0.9). Genomic fragment, which contains multiple nearby LGT genes derived from the same taxa, were further validated as a LGT event (representing a mobile genetic element or recombination event) via blasting to the origin genomic fragment of the origin taxa (overall similarity >85%).

### Bioinformatic analysis

Functional profile of each *A. muciniphila* strain was generated using the number of genes that were annotated into the same function category (i.e. the KEGG pathway or CAZymes). And Principal component analysis (PCA) was performed on the functional profiles of all *A. muciniphila* strains and visualized using the “ade4” package based on the R platform. For the comparative analysis, the stratification of age, gender, and BMI was adjusted based on the Generalized Linear Model (GLM, implemented in the R platform). Age and BMI were converted to categorical variables when performing the adjustment.

## Additional files

Additional file 1: Table S1. Isolation, sequencing, assembly and gene prediction information of 39 *A. muciniphila* isolates and strain ATCC BAA-835. (XLSX 16 kb)

Additional file 2: Figure S1. Pangenome “openness” of *A. muciniphila*. **Figure S2.** Principal components analysis shows the clustering of *A. muciniphila* isolates. **Figure S3.** Functional composition of the *A. muciniphila* pangenome. **Figure S4.** Principal components analysis on CAZymes profiles of *A. muciniphila* isolates. **Figure S5.** Relative abundance of *A. muciniphila* phylogroups in 368 Chinese gut microbiome samples. **Figure S6.** Gene flow and recombination events in *A. muciniphila* phylogroups. **Figure S7.** Loss and gain of orthologous groups during *A. muciniphila* evolution. (DOCX 682 kb)

Additional file 3: Table S2. Genome information of the 106 constructed *A. muciniphila* MAGs which are assembled from human and other mammalian gut microbiotas. (XLSX 17 kb)

Additional file 4: Table S3. Candidate horizontal gene transfer events identified from *A. muciniphila* genomes. (XLSX 11 kb)

## Abbreviations

ANI: average nucleotide identity
BMI: body mass index
CAZymes: carbohydrate-active enzymes
eggNOG: evolutionary genealogy of genes: non-supervised orthologous groups
KEGG: Kyoto encyclopedia of genes and genomes
LGT: lateral gene transfer
MAG: metagenome assembled genome;
PCA: principal components analysis
SNP: single nucleotide polymorphism
