# Supplementary material for: Genome sequencing of 39 Akkermansia muciniphila isolates reveals its population structure, genomic and functional diverisity, and global distribution in mammalian gut microbiotas
Source: BMC Genomics. 2017 Oct 18;18:800. doi: 10.1186/s12864-017-4195-3 (PMC5648452; doi:10.1186/s12864-017-4195-3)
Supplement: Supplementary file 1 — Isolation, sequencing, assembly and gene prediction information of 39 A. muciniphila isolates and strain ATCC BAA-835. (XLSX 16 kb) [file 12864_2017_4195_MOESM1_ESM.docx]

**Supplementary Figures**


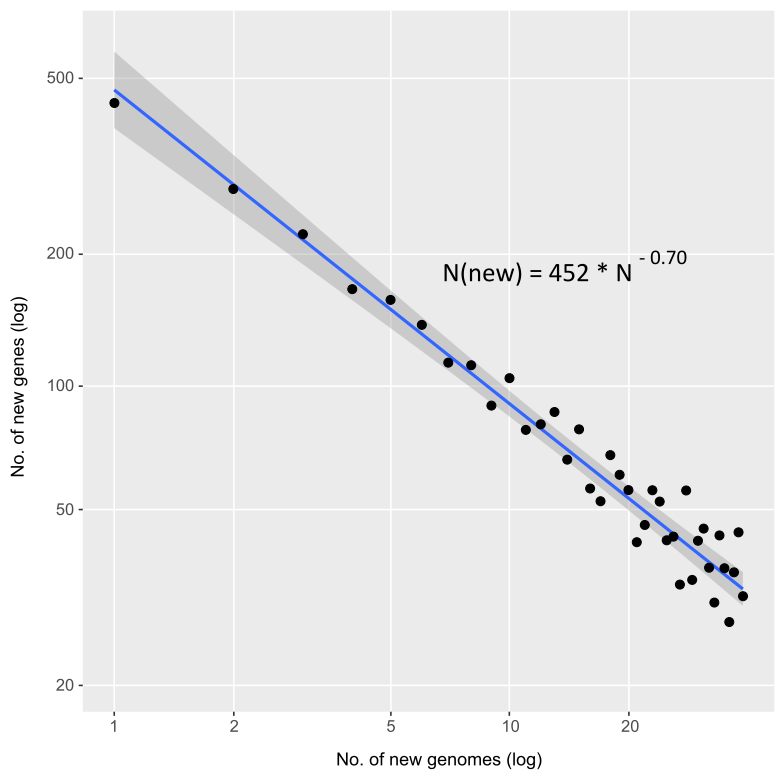


**Figure S1 | Pangenome “openness” of *A. muciniphila*.**

The black points indicate the number of new genes added to the total pangenome size as new genomes are added. The fitted line represents the power low, and the exponent ≥ -1 indicates an open state and that the category is boundless so new genes are discovered continually as new genomes are sequenced.


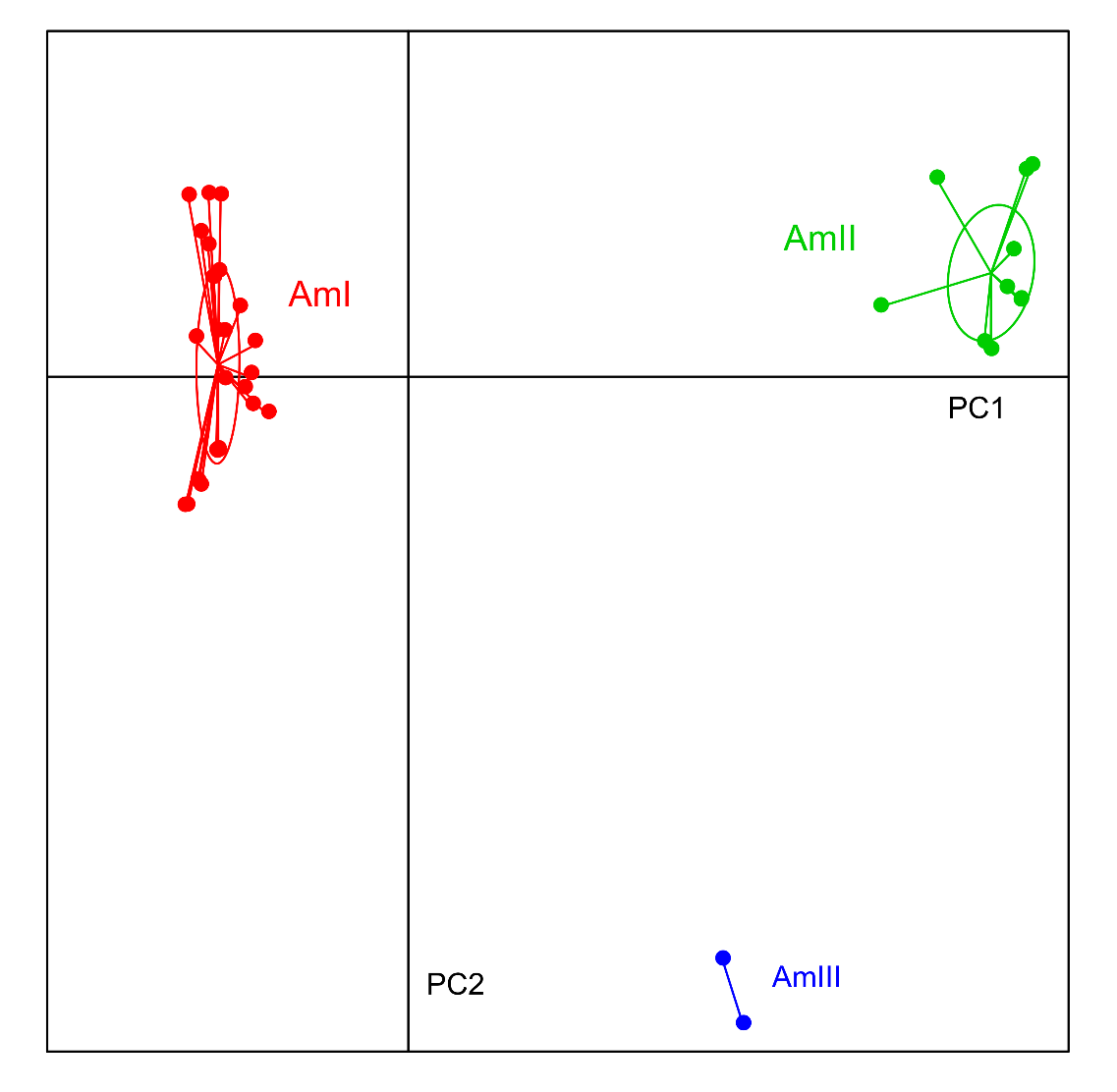


**Figure S2 | Principal components analysis shows the clustering of *A. muciniphila* isolates.**

PCA analysis are preformed based on the presence of accessory genes. The first two principal components (PC1 and PC2) are shown. Nodes represent the *A. muciniphila* isolates. Lines connect the isolates in the same phylogroup, and coloured circles cover the isolates near the center of gravity for each group.


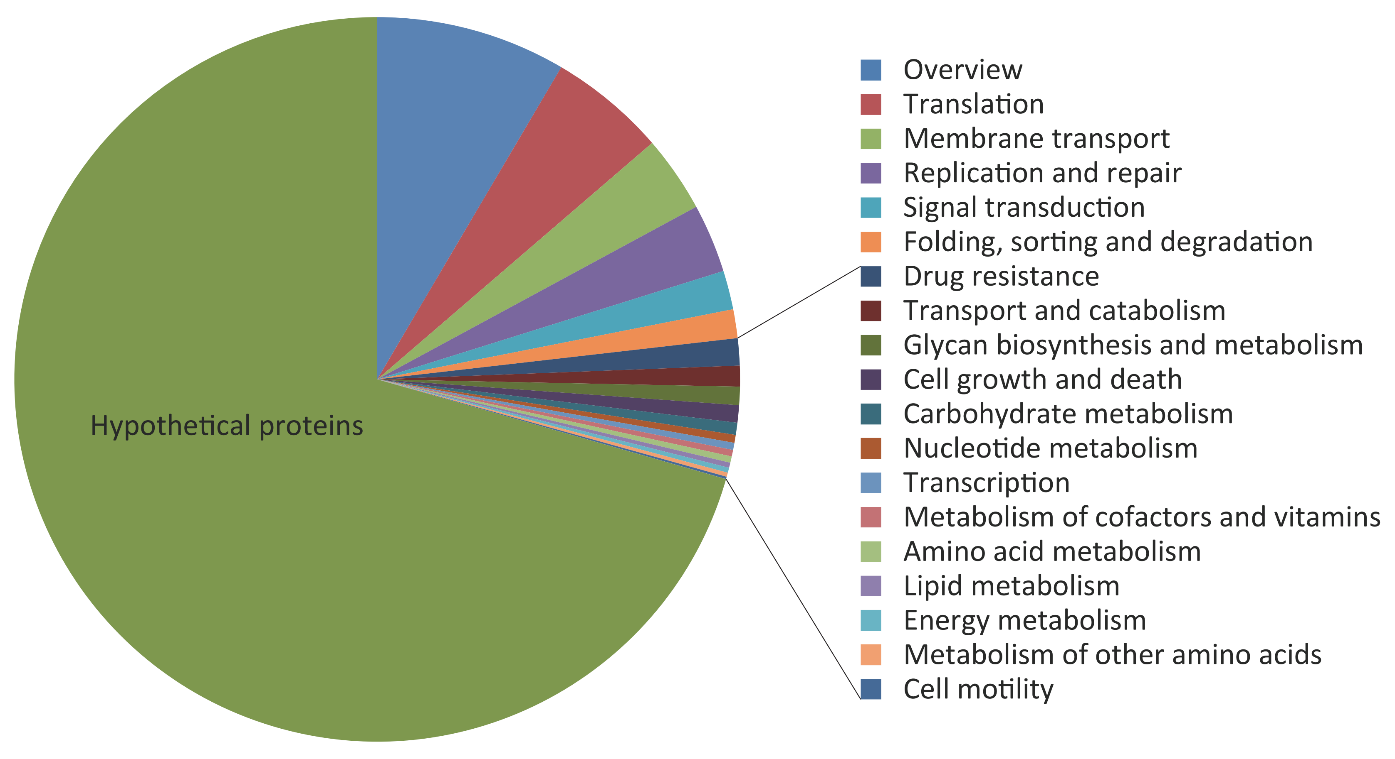


**Figure S3 | Functional composition of the *A. muciniphila* pangenome.**

The composition of KEGG pathways (at level 2) of 5,644 proteins in the *A. muciniphila* pangenome are shown.


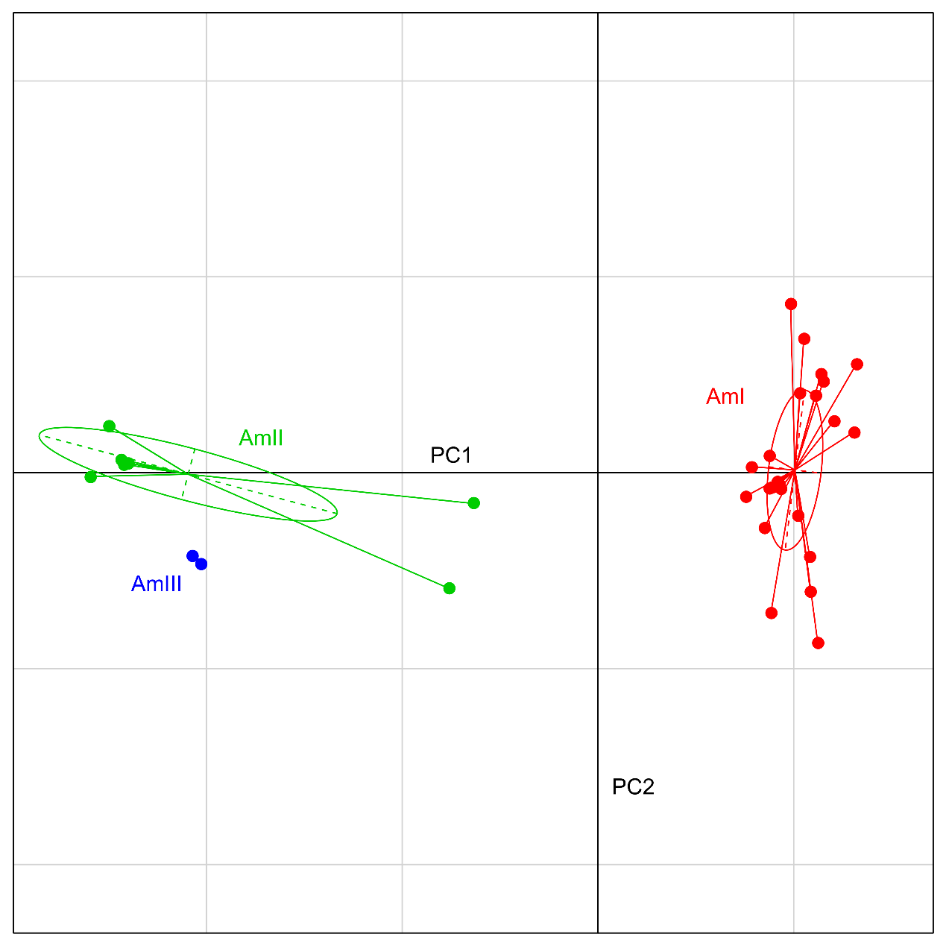


**Figure S4 | Principal components analysis on CAZymes profiles of *A. muciniphila* isolates.**

The first two principal components (PC1 and PC2) are shown. Nodes represent the *A. muciniphila* isolates. Lines connect the isolates in the same phylogroup, and coloured circles cover the isolates near the center of gravity for each group.


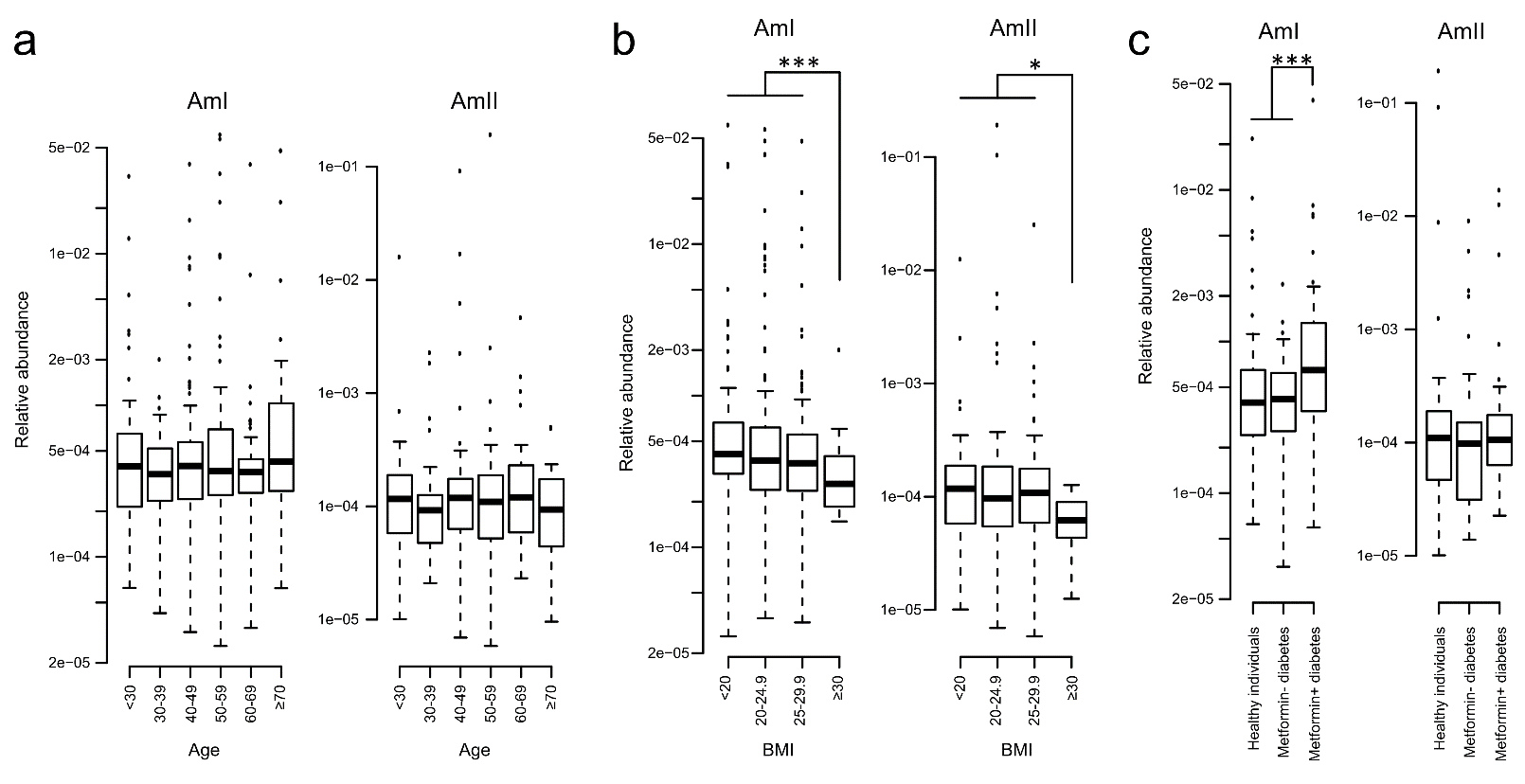


**Figure S5 | Relative abundance of *A. muciniphila* phylogroups in 368 Chinese gut microbiome samples.**

(**a**) Samples are grouped by age. (**b**) Samples are grouped by BMI. (**c**) Samples are grouped by healthy controls (n = 187), metformin untreated individuals (n = 91) and metformin treated individuals (n = 36). For **c**, some individuals with unknown drug information are removed. ‘*’ denotes *P* <0.05; ‘**’ denotes *P* <0.01; ‘***’ denotes *P* <0.001.


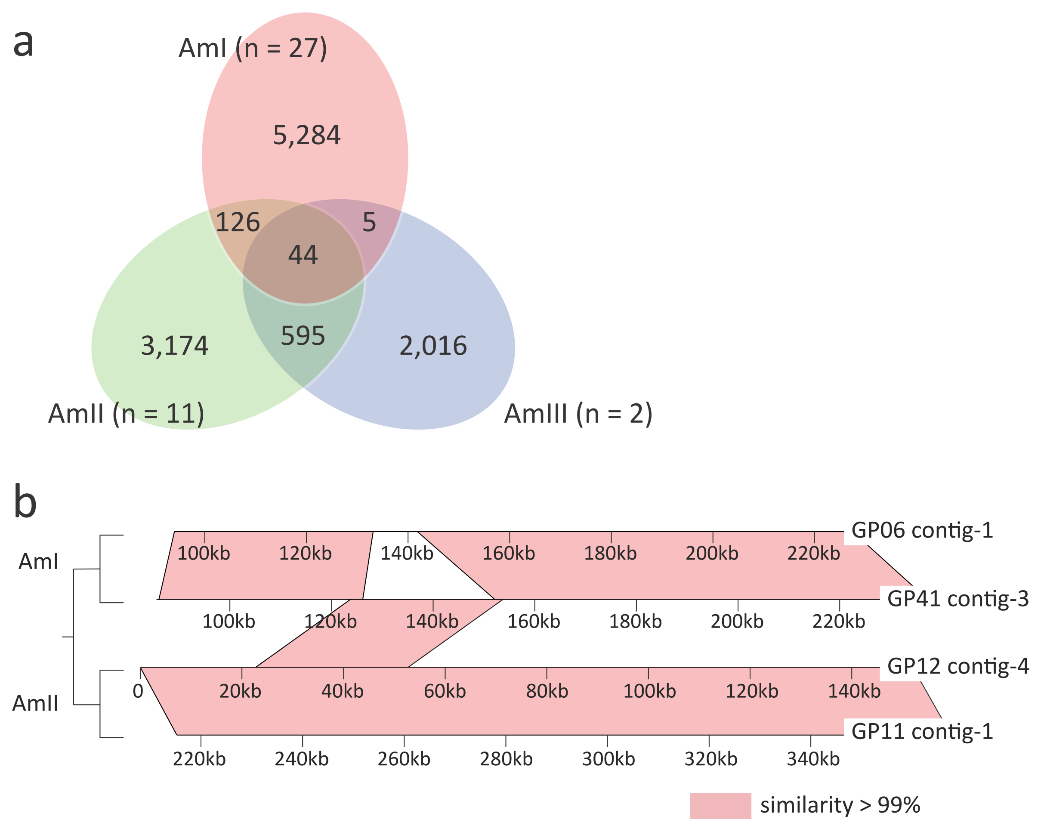


**Figure S6 | Gene flow and recombination events in *A. muciniphila* phylogroups.**

(**a**) Sharing of high identity genes between three *A. muciniphila* phylogroups. 770 genes that potential exchange (minimum nucleotide similarity, 95%) in two or more phylogroups are shown. (**b**) A 33 kbp region of homologous recombination between *A. muciniphila* GP41 (AmI) and other AmII strains.


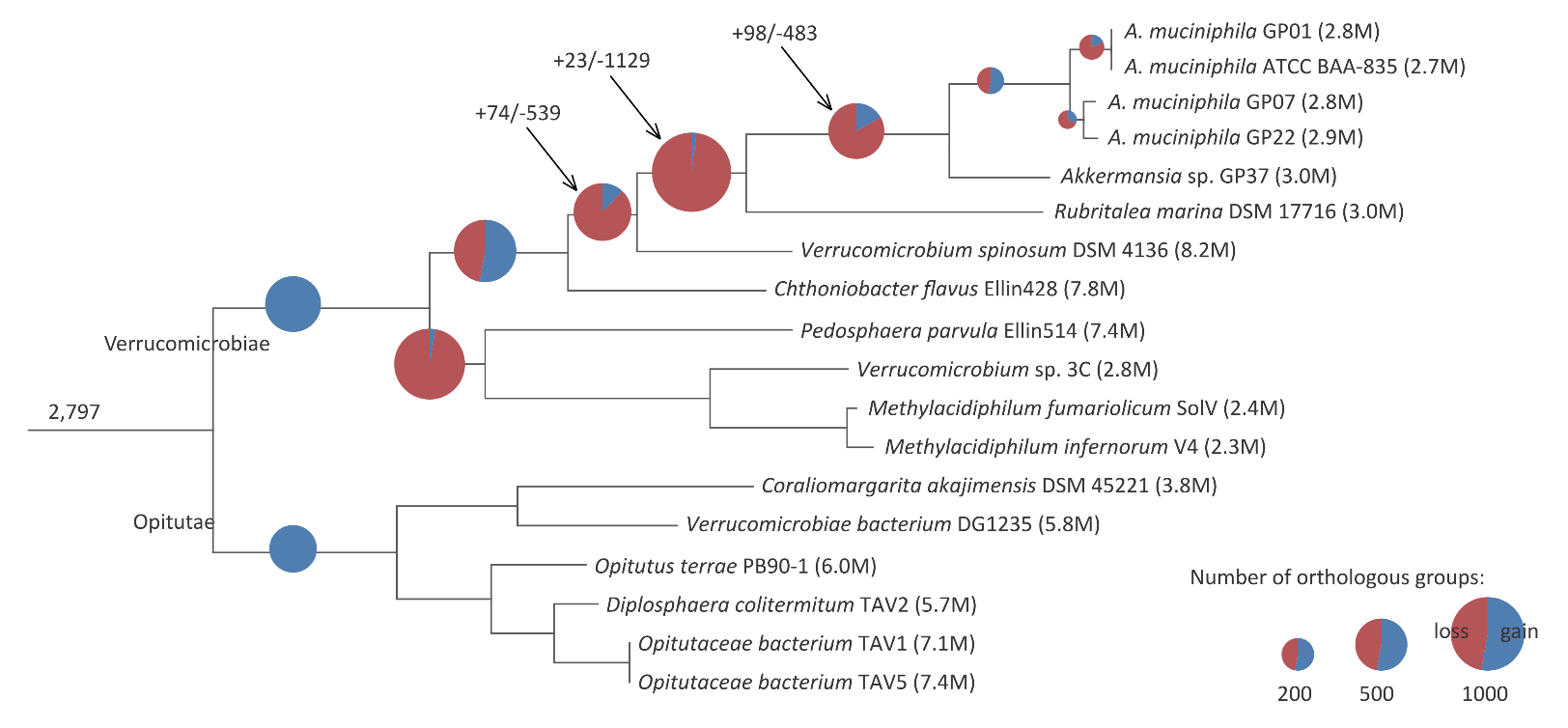


**Figure S7 | Loss and gain of orthologous groups during *A. muciniphila* evolution.**

Phylogenetic tree represents the evolution history of Verrucomicrobia phylum based on 18 NCBI available genomes. The area of a red or blue section in a pie is proportional to the number of lost or gained eggNOG orthogroups, respectively. Count (Csuros, 2010) software based on Dollo parsimony was used to reconstructing orthologous group contents at observed species and hypothetical ancestors, and orthologous group gains and losses at branching points.

Csuros, M. (2010) *Count: evolutionary analysis of phylogenetic profiles with parsimony and likelihood*. Bioinformatics 26: 1910-1912.
